# Effects of a High-Volume 7-Week Pectoralis Muscle Stretching Training on Muscle Function and Muscle Stiffness

**DOI:** 10.1186/s40798-023-00582-8

**Published:** 2023-06-01

**Authors:** Marina Reiner, Anna Gabriel, Daniel Sommer, Daniel Bernsteiner, Markus Tilp, Andreas Konrad

**Affiliations:** 1 Institute of Human Movement Science, Sport and Health, University of Graz, Mozartgasse 14, 8010 Graz, Austria; 2 Professorship of Conservative and Rehabilitative Orthopedics, Department of Sport and Health Science, Technical University of Munich, Munich, Germany

**Keywords:** Static stretching, Thoracic muscles, Tissue stiffness, Joint flexibility, Muscle strength, Long-term stretching

## Abstract

**Background:**

There is evidence that high-volume static stretching training of the lower limbs can increase the range of motion (ROM) while decreasing muscles stiffness. However, to date, there is no evidence on the effects of upper limb stretching training or its effect mechanism. Therefore, this study aimed to investigate the effects of a comprehensive 7-week static stretching training program of the pectoralis major muscle (PMa) on glenohumeral joint ROM, muscle force, and muscle stiffness.

**Methods:**

Thirty-eight healthy, physically active participants (23 male, 15 female) were randomly assigned to either the PMa-static stretching intervention (PMa-SS) group or the control group. The PMa-SS group performed a 7-week intervention comprising three sessions a week for 15 min per session, including three static stretching exercises of the PMa for 5 min each. Before and after the intervention period, shoulder extension ROM, muscle stiffness of the PMa (pars clavicularis), and maximal voluntary isometric contraction (MVIC) peak torque (evaluated at both long (MVIC_long_) and short (MVIC_short_) muscle lengths) were investigated on a custom-made testing device at 45° shoulder abduction.

**Results:**

In the PMa-SS group, the shoulder extension ROM (+ 6%; *p* < 0.01; *d* = 0.92) and the MVIC_long_ (+ 11%; *p* = 0.01; *d* = 0.76) increased. However, there were no significant changes in MVIC_short_ or in PMa muscle stiffness in the PMa-SS group. In the control group, no changes occurred in any parameter.

**Conclusion:**

In addition to the increase in ROM, we also observed an improved MVIC at longer but not shorter muscle lengths. This potentially indicates an increase in fascicle length, and hence a likely increase in sarcomeres in series.

## Key Points

- This was the first study which investigated the effects of a comprehensive 7-week static stretching training program of the pectoralis major muscle on glenohumeral joint ROM, muscle force, and muscle stiffness.
- The stretching group showed an increase in ROM and MVIC at longer muscle lengths
- The increase in ROM was not associated with a decrease in muscle stiffness and hence, an increased stretch tolerance and/or changes in other structures such as tendons, ligaments, or capsules might be responsible for the increase in ROM

## Introduction

From an anatomical perspective, the shoulder joints are the most flexible joints of the human body [1]. For this reason, surrounding muscles and other connective tissue structures play an essential role in proper joint positioning, function, and performance [2]. One major muscle group of the shoulder joint is comprised of the pectoralis muscles. The length, stiffness, and functioning of the pectoralis muscles, and mainly the pectoralis minor muscles, are believed to be associated with different restrictions of the glenohumeral joint, e.g., altered scapular kinematics, positioning, and general shoulder girdle posture, shoulder range of motion (ROM) deficits, and glenohumeral instability [2–7]. Although the connection between shoulder girdle posture, functional parameters, and musculoskeletal disease and pain is still controversial [8, 9], it is evident that muscle flexibility and sufficient joint mobility are fundamental for both health and performance. Especially in overhead and throwing sports, such as tennis, a glenohumeral internal rotation deficit can be present [4], and the risk of shoulder injury is therefore increased. Potential reasons for this deficit might be reduced muscle length or increased stiffness of the pectoralis muscles, or other soft tissue alterations [4, 8, 9].

Long-term static stretching training is a well-known technique that is able to induce changes in the ROM of a joint [10–13]. Some studies have even reported an improvement in muscle force after a comprehensive static stretching intervention [12, 14–16], while others have reported no such improvement [13, 17, 18]. A potential mechanism for the changes, especially in ROM, is a change in stretch/pain perception after extensive stretching programs such as 10 min stretching training per week for a period of 6 weeks [13, 19]. This mechanism is referred to as sensory theory [20]. However, if a comprehensive stretching approach with longer stretch durations (e.g., > 30 min per week) is applied, a decrease in muscle stiffness has been reported [11]. This finding may correspond to increased fascicle length [12]. However, most studies have investigated the effects of static stretching on the muscles in the lower extremities, while far less is known about the upper limbs, in particular the shoulder girdle complex [4]. To date, it is not clear if the findings of studies on the muscles in the lower limbs can be directly transferred to the muscles in the upper extremities. As an example foam rolling, which has shown similar effects on ROM [21, 22] and muscle performance [23], can induce acute changes in various parameters at the leg muscles [24, 25], while no changes were reported at the pectoralis major pars clavicularis muscle [26]. Consequently, it can be assumed that the tissues of the upper and lower limbs might react differently to similar long-term stretching stimuli. Hence, there is a need to investigate the effect of a long-term static stretching intervention on shoulder ROM and to identify the mechanical factors underpinning increased ROM, such as muscle stiffness. Targeting the pectoralis muscles seem justified because of the relation between glenohumeral flexion ROM and pectoralis minor length [9]. This might allow to get more information about the functioning of individual parts of the shoulder complex and how they are affected.

Therefore, the aim of this study was to investigate the effects of a comprehensive 7-week stretching program for the pectoralis muscles, performed 3 times per week for 15 min each session, on shoulder extension ROM, muscle stiffness (assessed with shear wave elastography), and maximal voluntary isometric contraction (MVIC) peak torque at two different arm positions, that induced either a long or short pectoralis major (PMa) muscle length. We hypothesized that the static stretching program would lead to an increase in shoulder extension ROM, a decrease in PMa muscle stiffness, and an increase in MVIC torque values.

## Materials and Methods

**Fig. 1:**
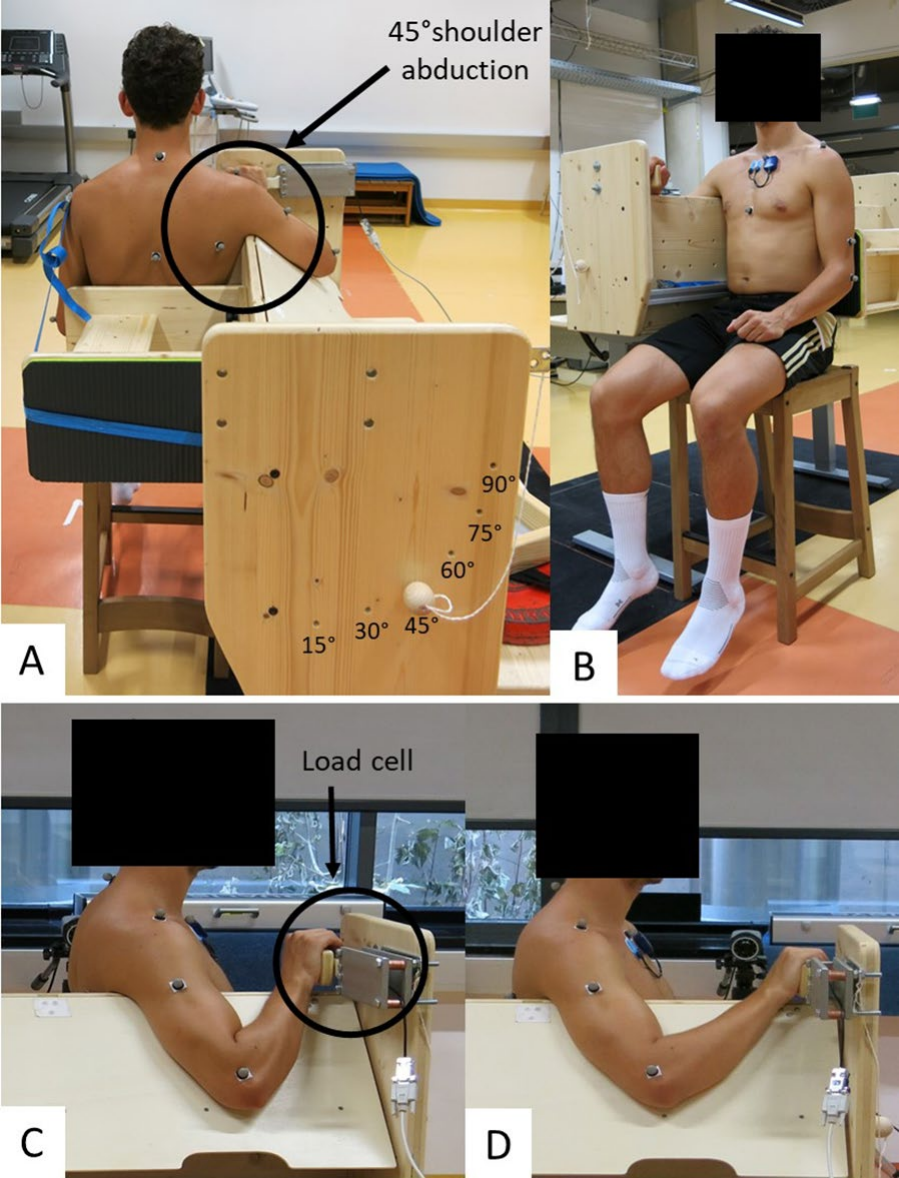
Custom-made testing device. When the participant sits upright on the chair included in the custom-made testing device, their back touches the moveable, individually fixable backrest (**B**), which allows us to adapt the distance between hand palm and trunk to reach 45° (**C**) or 90° (**D**) elbow flexion and a shoulder flexion of 8° ± 8.6° or 31° ± 7.5° (mean ± std) long or short muscle length, respectively. The device is adjustable in height and is positioned in a way such that the participant’s shoulders are parallel to the floor, while the top edge of the device is placed under the armpit (**A**). The relaxed arm rests on a moveable board fixed at 45° shoulder abduction (**A**). At the level of the shoulder joint, a load cell is placed on the front edge of the device, on which the participant places their palm (**C**, **D**)

**Fig. 2:**
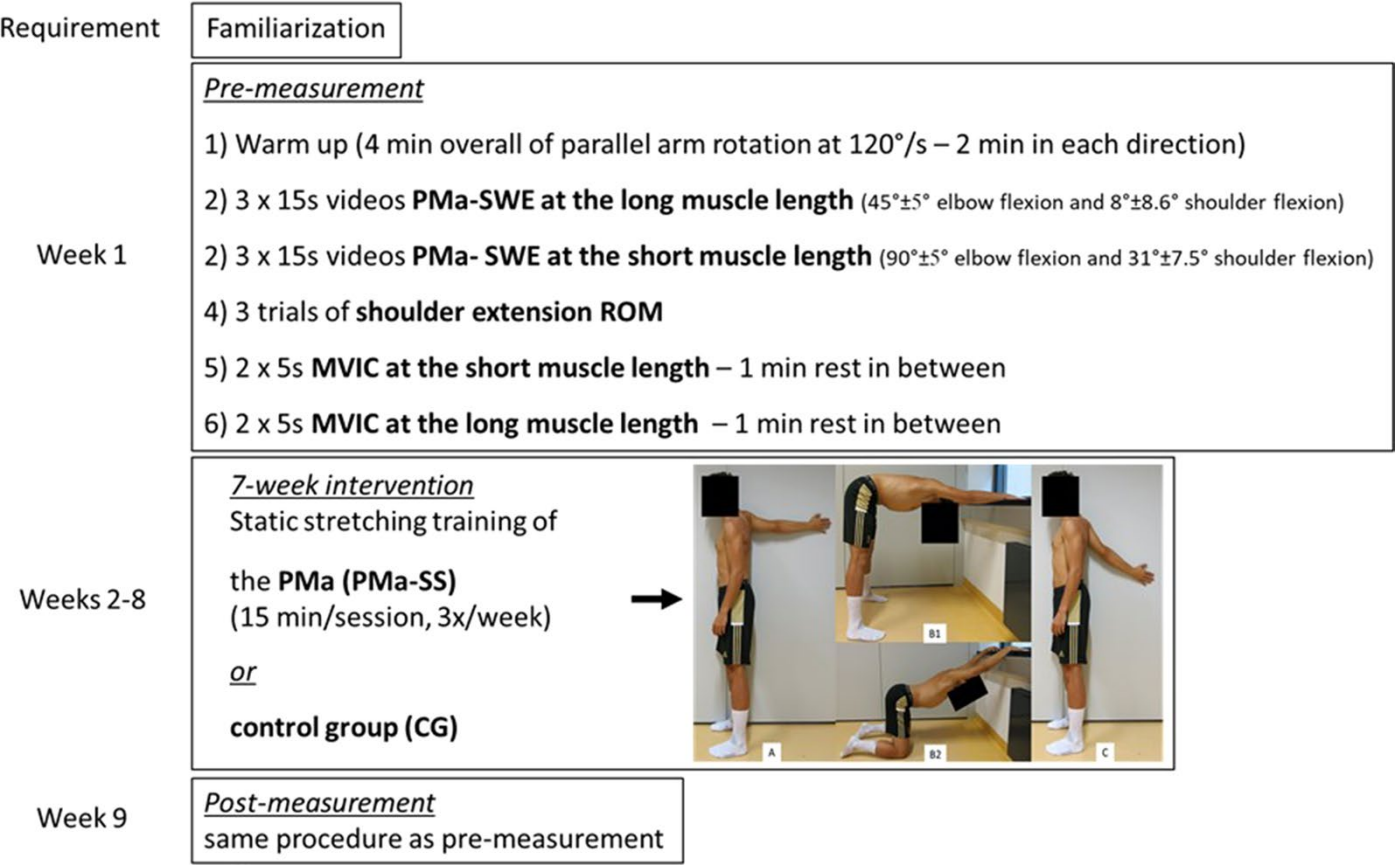
Schematic representation of the study design; PMa, pectoralis major; SWE, shear wave elastography; ROM, range of motion; MVIC, maximal voluntary isometric contraction; **A** stretching exercise 1; **B1/B2** stretching exercise 2 (two different options); **C** stretching exercise 3

Participants visited the laboratory on three occasions: for a familiarization session, a pre-intervention session (pre), and a post-intervention session (post) after the 7-week intervention period. By picking hidden cards they were assigned to either the intervention (PMa-SS) or the control group (CG). However, males and females were separately randomized to ensure equal distribution between groups. As a warm-up, each participant performed 4 min of synchronous arm rotations with extended elbow joints and the greatest radius possible (2 min in each direction, alternating direction every minute) at a speed of 120°/s (= 20 rotations per minute), which was standardized via metronome signals. The shear wave elastography (SWE) of the PMa, shoulder extension ROM, and MVIC peak torque were measured on the dominant arm (used for writing) in a sitting position at 45° shoulder abduction on a custom-made testing device (Fig. 1A). For the SWE and MVIC measurements, the participant was positioned with the hand on shoulder joint axis height in front of the body and flexed elbow at 90° ± 5° or 45° ± 5°. This induced a shoulder flexion angle of 31° ± 7.5° or 8° ± 8.6° (mean ± SD), which led to short or long PMa lengths, respectively (Fig. 1C + D). For the shoulder extension ROM measurement, the elbow flexion was 90°. The height of the testing device and the chair position were individually adjusted for every participant. The position was recorded during the pre-measurements, to ensure the same position during the post-measurements. Muscle activity was measured via surface electromyography (sEMG) on the medial part of the PMa during all the tests. The order of measurements was the same in all the tests (Fig. 2), to exclude any interfering effects between tests. The ethical committee of the University of Graz (approval code GZ. 39/4/63 ex 2021/22) approved the study, and it was conducted in accordance with the standards of the Declaration of Helsinki.

### Participants

In a previous study, we performed a 6-week static stretching training program on the triceps surae muscle, and we observed a large effect size (Cohen’s *d* = 1.1) for the increased ROM [13]. Hence, with the power to detect a large effect size, we calculated a minimum sample size of 15 participants for each group for this study (difference between two dependent means, effect size = 0.8, *α* = 0.05, 1 − *β* = 0.8) using G*Power software [27].

Thirty-eight healthy, physically active volunteers (average weekly training duration: 9.8 ± 5.5 h; males: 23, age: 26.4 ± 5.3 years, height: 183.3 ± 6.7 cm, and body mass: 81.9 ± 7.7 kg; females: 15, age: 28.4 ± 4.4 years, height: 167.9 ± 4.9 cm, and body mass: 62.9 ± 7.4 kg) participated in at least 80% of the training sessions during the 7-week intervention phase. All participants reported no previous injuries in the upper and lower extremities and were instructed to avoid exhausting training sessions in the 24 h before the measurements. All the procedures were explained to the participants, and each participant gave written informed consent before they were included in the study.

### Procedure

#### Shear Wave Elastography (SWE)

**Fig. 3:**
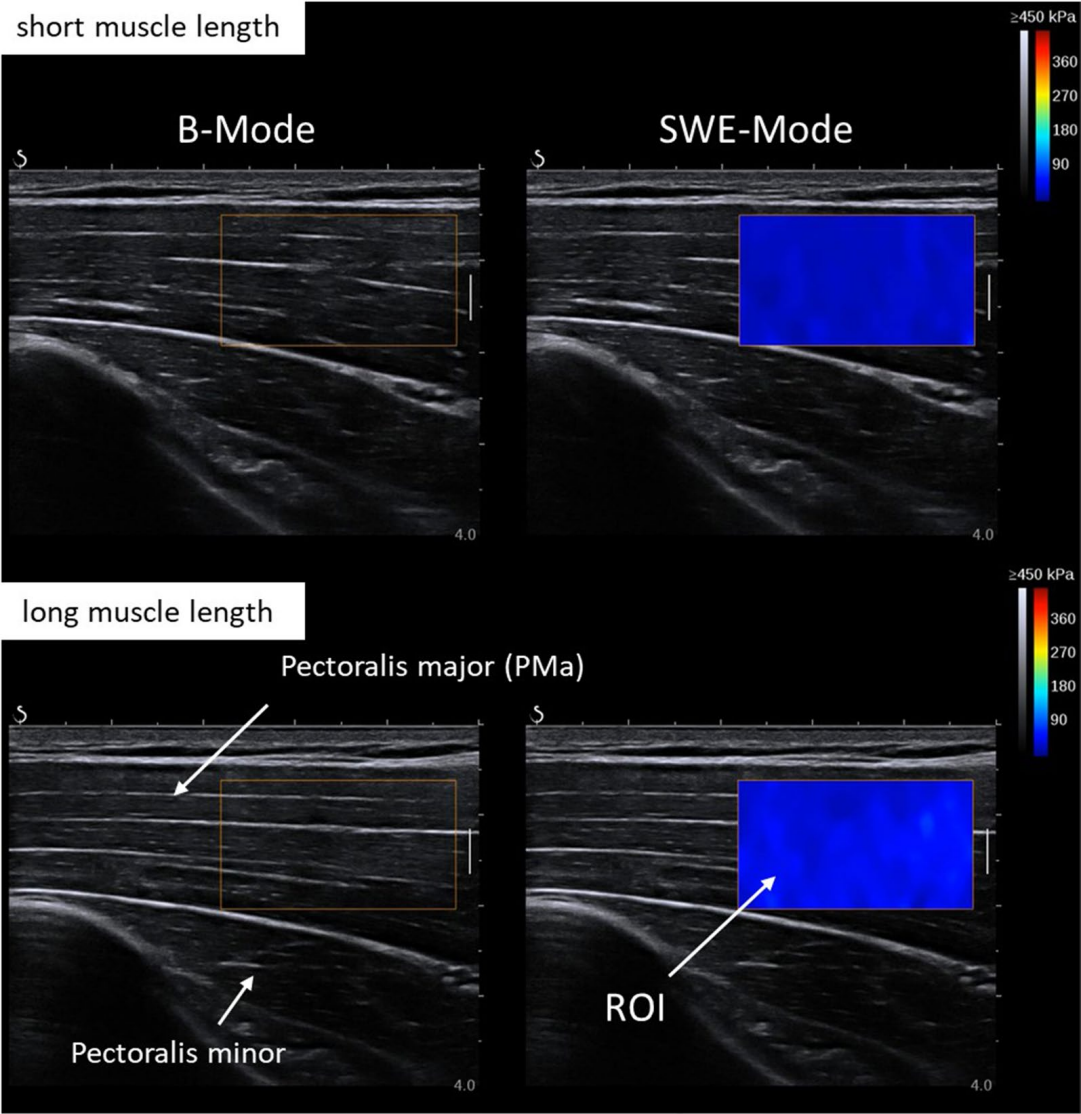
Example of an ultrasound of the pectoralis major (PMa) muscle (pars clavicularis) at a long (top) and short muscle length (bottom), in a relaxed state. The lighter blue in the ROI in the lower picture signalizes a higher muscle stiffness, as it occurs in the longer muscle length. SWE, shear wave elastography; PMa, pectoralis major; ROI, region of interest

An ultrasound scanner (Aixplorer V12.3, Supersonic Imaging, Aix-en Provence, France) was coupled with a linear transducer array (4–15 MHz, SuperLinear 15-4, Vermont, Tours, France) and used in SWE mode (musculoskeletal preset, penetration mode, smoothing level 5, persistence off, scale 0–450 kPa). For better orientation and reliability, the measurement position of the most proximal part of the PMa (pars clavicularis), half-way between the sternomanubrial joint and the beginning of the axillary fold [28], was marked on the skin, while the participant stood upright with relaxed arms. Muscle stiffness, measured with shear wave velocity as an index for tissue extensibility, was measured with a hand-held technique [29] in the 45° relaxed shoulder abduction position, as shown in Fig. 1, at long muscle length (SWE_long_, Fig. 1C), and at short muscle length (SWE_short_, Fig. 1D). During the measurements, a B-mode picture of the first attempt (i.e., the first assessment of the pre-measurement) was used for visual support. The ultrasound probe was aligned in plane with the muscle fascicles, and the region of interest (ROI) was defined as large as possible, in between the PMa muscle aponeuroses (Fig. 3). During the measurements, the resting state of the muscle was controlled by observing the sEMG signals. Three SWE videos of 15 s each were recorded at the marked skin position in both positions (i.e., short and long muscle lengths). Furthermore, the mean of five consecutive frames with the lowest standard deviation within the ROI (averaged values, analyzed with MATLAB R2017b, MathWorks, Natick, USA, [30]) within one video was determined [30]. Finally, the muscle stiffness was calculated as the mean between the two closest mean values of the three videos [30].

#### Shoulder Extension Range of Motion (ROM)

For testing the ROM, a 3D-motion capture system (Qualisys, Gothenburg, Sweden) was used, with the participant sitting next to the custom-made device. Sixteen reflective markers (1 cm diameter) were positioned according to the Qualisys “CAST upper body marker set” and extended with the markers of the “CGM upper body marker set” on the participant’s arms and trunk. To ensure that the shoulder movement was conducted without changes of elbow joint angle, a custom-made fixation with an angle of 90° was used, which was strapped to the participant’s arm. Moreover, the participant’s trunk was fixed with a strap to the backrest, to avoid accessory movements. The test was started in a neutral shoulder joint position, and the participant was asked to move the fixed elbow along the 45° fixed board as far behind the body as possible, without pulling the shoulder to the neck. The movement was performed slowly and repeated three times, with 15 s breaks in between. The sEMG of the PMa was recorded, and if any activity was detected, the trial was repeated. The recorded markers were mapped in a model consisting of the upper arm and the torso. The angles of the shoulder joint were extracted using Visual3D Professional × 64 (C-Motion, Inc., Germantown, Virginia, USA). The relations of the torso and the upper arm positions to each other were used to calculate the joint angles in all three planes of motion. The attempt with the highest shoulder extension ROM was considered for further analysis.

#### Maximal Voluntary Isometric Contraction (MVIC) Peak Torque

MVIC testing was performed twice in both arm positions, at the long (MVIC_long_) and short (MVIC_short_) muscle lengths. The participant was seated as described in Fig. 1, with their palm on the load cell (VPG Force Sensors, Model 1022, Tedea - Huntleigh, Netanya, Israel) and fingers relaxed. To avoid any co-activation in the rest of the body, the participant was asked to let their legs hang loosely while sitting on the chair. After a verbal countdown for preparation, the participant was asked to push as hard as possible against the load cell for 5 s. Verbal encouragement was given during all attempts. Between each attempt, there was a 1-min break to avoid fatigue [13]. The data were recorded with a sampling rate of 1000 Hz, while they were applied to the program of the 3D camera system via an amplifier. Further, the load was calculated with a conversion factor between units (Volts (V) to Newton (N)), determined during the calibration process with a 10 kg weight. The mean of the two MVIC peak values was taken for further analysis.

#### Surface Electromyography (sEMG)

Muscle activity was measured during all measurements (SWE, ROM, and MVIC) using sEMG (myon 320, myon AG, Zurich, Switzerland) with a sampling rate of 2000 Hz. The skin was prepared and the 2 cm diameter surface electrodes (Blue Sensor N, Ambu, A/S, Ballerup, Denmark) were placed with an interelectrode distance of 2 cm on the most medial part of the PMa (pars clavicularis) of the dominant arm, to ensure a proper ultrasound probe positioning in the middle of the PMa muscle. If required, the sEMG signals of the MVIC trials were high pass filtered (10 Hz, Butterworth) and the root mean square (RMS, 50 ms window) was computed. If any muscle activity of the PMa was detected visually in the sEMG during ROM or SWE testing, the trial was repeated. Values below 5% of muscle activity recorded during MVIC were defined as *relaxed state* [31].

### Static Stretching Intervention

The participants of the PMa-SS group were asked to perform the static stretching training, consisting of three exercises for 7 weeks, three times a week (recommendation: Monday, Wednesday, Friday). Each exercise was performed continuously for 5 min, resulting in an overall stretch duration of 15 min per session. The stretching exercises were coordinated with a physiotherapist and selected to target the pectoralis muscles. For Stretching exercise 1 (Fig. 1A), the extended arm was positioned at a wall at shoulder height behind the body, with the palm facing the wall. The stretching intensity was adjusted by rotating the trunk transversely, toward or away from the wall. Stretching exercise 2 was a bilateral stretching exercise, where the hands were placed between breast and hip height on the wall or on an object. The hip was flexed, the arms were kept extended, and the shoulders were moved downwards to the floor (Fig. 1B1). For increased comfort, the participant was allowed to perform the exercise in a kneeling position (Fig. 1B2). Stretching exercise 3 was performed similarly to Stretching exercise 1, with the difference being that the arm was held at an angle of 45° in relation to the ground, with the palm facing the wall (Fig. 1C). For all three stretching exercises, the participant was instructed to maintain the stretch intensity at the point of discomfort during the whole stretch duration. Stretching exercises 1 and 3 were performed with the dominant arm, but the participants had the choice to do these exercises bilaterally.

Participants of the control group performed an alternative training program of the structures of the lower leg (rolling and stretching of the sole of the foot) to the same extent as the PMa-SS group.

### Statistical Analysis

The statistical analysis was performed using SPSS (version 28, SPSS Inc., Chicago, Illinois). We tested all parameters for normal distribution with the Shapiro–Wilk test. If the data were normally distributed (such as shoulder extension ROM, MVIC_long_, MVIC_short_), a linear mixed model ANOVA (within factor: time (pre vs. post) and between factor: group (intervention vs control)) was calculated. If there were significant interaction effects in the ANOVA, a paired *t* test was performed. For nonparametric data (such as SWE_short_, SWE_long_), we applied the Wilcoxon test (between the pre- and post-data within each group) instead. Following a significant effect in the Wilcoxon test, a Mann–Whitney *U*-test between the delta values of the two groups was performed. Each participant’s data at baseline were tested for similarities between groups using a *t* test (if normally distributed) or a Wilcoxon test (if not normally distributed). The inter- and intra-day intraclass correlation coefficients (ICC, 2-way mixed effects model, absolute agreement definition) were calculated for the SWE measurements in both arm positions. The inter-day ICC was calculated between the familiarization session and the first measurement. The standard error of the measurement of the muscle stiffness values was determined as the standard deviation multiplied by the square root of one minus the ICC. Cohen’s *d* was calculated, and the effect size was defined as 0.2, 0.5, and 0.8 for a small, medium, and large effect size, respectively, according to Cohen [32]. The alpha level was set to 0.05.

## Results

### Baseline Measurement Quality and SWE Reliability

No significant differences were detected in the pre-measurement data of the stretching and control groups for shoulder extension ROM (*p* = 0.88), MVIC_long_ (*p* = 0.54), MVIC_short_ (*p* = 0.63), SWE_long_ (*p* = 0.51), and SWE_short_ (*p* = 0.2). The SWE intra-day ICC values for the PMa at long and short muscle length were 0.98 (0.91–0.99) and 0.99 (0.98–0.99), respectively. Additionally, the SWE coefficient of variance (CV) at short and long muscle length was 2.8 ± 2.6 and 5.1 ± 5.2, respectively. The SWE inter-day ICC values for the PMa at long and short muscle length were 0.96 (0.78–0.99) and 0.89 (0.42–0.98), respectively.

### Muscle Stiffness Values

The Wilcoxon test showed no significant change in muscle stiffness of the PMa between the pre- and post-values at long (SWE_long_) and short (SWE_short_) muscle length, respectively, in both the PMa-SS group (*p* = 0.29 and *p* = 0.36) and control group (*p* = 0.59 and *p* = 0.53).

### Range of Motion (ROM)

For the shoulder extension ROM measurements, significant time (*p* = 0.01; *F* = 7.21) and interaction effects (*p* < 0.01; *F* = 10.97) were detected. A pairwise comparison showed a significant increase in the PMa-SS group (*p* < 0.01, *d* = 0.92), but not in the control group (*p* = 0.64; *d* = 0.11).

### Maximal Voluntary Isometric Contraction (MVIC) Peak Torque

The mixed factorial ANOVA for the MVIC_long_ measurements revealed significant time (*p* = 0.02; *F* = 5.93) and interaction effects (*p* = 0.01; *F* = 6.81). The pairwise comparisons showed a significant increase in the PMa-SS group (*p* = 0.01; *d* = 0.76), while there was no significant change in the control group (*p* = 0.89; *d* = 0.03).

The mixed factorial ANOVA for the MVIC_short_ data revealed no significant time (*p* = 0.38; *F* = 0.79) or interaction (*p* = 0.11; *F* = 2.77) effects.

All the mean pre- and post-values of the parameters are presented in Table 1.

**Table 1:** Absolute values of all the tested variables before and after the 7-week static stretching intervention

	Intervention group (PMa-SS)		Control group (CG)		Effect size	
	PRE	POST	PRE	POST	PMa-SS	CG
	Mean ± SD	Mean ± SD	Mean ± SD	Mean ± SD		
Shoulder extension ROM (°)	68.6 ± 6.2	73.1* ± 7.7	68.5 ± 7.6	69.1 ± 5.6	*d* = 0.9	*d* = 0.1
MVIC at long muscle length (N)	288.9 ± 118.4	332.4* ± 117.0	267.5 ± 97.3	258.5 ± 100.3	*d* = 0.8	*d* = 0.03
MVIC at short muscle length (N)	339.3 ± 127.0	365.6 ± 124.7	320.0 ± 114.4	301.8 ± 118.0	*F* (group) = 0.8 *F* (interaction) = 2.8	
Muscle stiffness at long muscle length (kPa)	9.9 ± 4.1	8.6 ± 2.4	10.4 ± 3.6	11.3 ± 4.6	*p* = 0.3	*p* = 0.6
Muscle stiffness at short muscle length (kPa)	7.5 ± 2.5	6.5 ± 2.1	7.3 ± 2.4	8.5 ± 5.1	*p* = 0.4	*p* = 0.5

ROM, range of motion; MVIC, maximum voluntary isometric contraction peak torque; PMa-SS, pectoralis major static stretching training

*Significant difference between pre- and post-values; *α*-level = 0.05

## Discussion

The aim of this study was to investigate the effects of a comprehensive 7-week static stretching program of the pectoralis muscles on shoulder extension ROM, muscle strength, and muscle stiffness of the PMa in two different arm positions (long and short muscle length). The results showed a significant increase in shoulder extension ROM and increased MVIC peak torque at the long muscle length after the static stretching intervention, while the MVIC peak torque at the short muscle length did not change. Moreover, no changes were detected in the muscle stiffness of the PMa in either of the tested positions (i.e., long and short muscle length).

As expected, the shoulder extension ROM increased significantly after the 7-week static stretching intervention. Several other studies on the effects of long-term static stretching interventions on the lower limbs have reported a significant increase in joint flexibility, independent of the stretch duration or stretch intensity [10, 12, 13, 33, 34]. Thomas et al. [33] even showed in their review that a minimum stretching time of 5 min per week increases joint ROM. Although such a minimum amount of stretch duration can increase the ROM, Freitas et al. [19] argued that a low-volume stretching intervention might not be large enough stimulus to induce changes in the structural parameters of the muscle–tendon unit (i.e., stiffness, fascicle length). In contrast, recent studies that applied high-volume stretching training (i.e., > 30 min of stretching per week) have reported changes in the muscle stiffness [11, 12], fascicle length, and muscle cross-sectional area [12]. Therefore, in the current project, an extensive stretching duration for the pectoralis muscles of 45 min per week was applied. Our hypothesis was that the increase in ROM could be explained by a less stiff PMa muscle tissue. In the current study, no changes in muscle stiffness of the PMa muscle were detected, and hence, the increased ROM in the PMa-SS group might be due to the increase in stretch tolerance, i.e., reduced pain sensitivity [20]. However, since the shoulder joint has multiple degrees of freedom and has more muscles involved than the PMa, the stiffness of the other muscles (e.g., pectoralis minor) or other structures such as tendons, ligaments, or capsules might have contributed to the increased ROM detected in the current study.

Concerning muscle force, we found an increase in MVIC at the long muscle length (45° ± 5° elbow flexion and 8° ± 8.6° shoulder flexion), but not at the shorter muscle length (90° ± 5° elbow flexion and 31° ± 7.5° shoulder flexion). Thus, it is possible that the high stretch duration induces a change in muscle tissue that allows increased muscle force in the lengthened muscle state. This finding goes in line with the results of acute stretching studies [35–37]. McHugh & Nesse [36] reported a loss in isometric muscle strength in a shortened, but not in a lengthened muscle state in the hamstring muscles following an acute static stretching intervention (6 × 90 s) [36]. Additionally, Weir et al. [37] stretched the plantar flexors for 5 × 120 s. The MVIC values conducted in the same angle as pre-intervention were decreased, but in a longer muscle length no such decrease was reported [37]. Behm et al. [38] summarized in their review, that acute and long-term stretching interventions might induce a shift of the active muscle length-tension relation toward the descending curve and longer muscle lengths [38]. Another potential explanation could be the increase in sarcomeres in series due to static stretching, as this has been observed in some animal studies [39–41]. Data from Table 1 indicate that the PMa is working in the decreasing part of its force–length relationship (with higher forces at shorter muscle lengths, i.e., shorter sarcomeres). An increase in the serial number of sarcomeres would therefore lead to shorter sarcomeres at a given muscle length, and therefore to increased force production, as seen in our results. However, there are controversial findings regarding the effects of long-term static stretching interventions on muscle architecture (e.g., fascicle length, pennation angle, and muscle thickness) in humans. Sato et al*.* [42] did not detect any changes in muscle architecture after a 6-week stretching intervention (6 min per week). On the other hand, Simpson et al. [43] were able to detect increased fascicle length and muscle thickness after a 6-week static stretching intervention (15 min per week), but these effects did not affect muscle performance. Freitas and Mil-Homens [44] detected increased fascicle length and an increase in knee extension ROM after 8 weeks of static stretching (37.5 min per week). In the present study, detailed muscle architecture measurements were not performed, but muscle stiffness was quantified. The weekly training duration of 45 min resulted in increased muscle strength in the longer muscle and increased shoulder extension ROM, which may indicate changes in the fascicle length of the PMa. Possible changes in the muscle architecture might be explained by the high stretch duration and intensity (stretching to the point of discomfort). Taking in account that several tissues are working together to move the shoulder joint [45], it might need to be necessary to consider data of more structures to be able to find an explanation for changes in force-producing tasks in a flexed shoulder position.

To date, the majority of chronic stretching studies have been concerned with the lower limbs and included healthy participants, while chronic stretching studies of the upper limbs have mostly involved symptomatic participants with different physical issues. Kim et al. [2] stretched the pectoralis minor muscle (25 min per week for 4 weeks) in participants with rounded shoulder posture and reported an increase in extension and horizontal abduction strength. Even though the stretching duration was shorter than in the present study, increased shoulder muscle strength was reported, as seen in the present study. The study by Rosa et al. [46] reported decreased shoulder pain and decreased disability in the shoulder, arm, and hand in shoulder pain patients after a 6-week stretching program (28 min per week), while no changes were seen in pectoralis muscle length in healthy or symptomatic participants. Therefore, the authors assumed that muscle length might not be relevant to induce pain reductions or improvements in function [46]. The results of the present study show that shoulder flexibility can be increased in healthy participants, probably due to the longer weekly stretching duration (45 min vs. 28 min).

Although we did not include patients in our studies, the results might also be clinically relevant for the treatment and prevention of various pathologies. Some studies have reported decreased shoulder mobility and strength, increased risk of injury (especially in overhead athletes), and many other complications to be associated with an altered shoulder girdle complex position, often resulting from increased muscle stiffness [2, 5–8]. Currently, there is no clear evidence whether scapula, shoulder joint, or even general posture exercises promote rehabilitation for musculoskeletal problems (e.g., subacromial impingement syndrome) [47]. Nevertheless, there are several pathologies for which treatment methods for the PMa would appear to be uniquely useful. It is recommended for overhead sports, for example, to avoid glenohumeral ROM deficits by performing regular mobility and stretching exercises [8].

However, to date, little is known about the long-term effects of upper limb static stretching in overhead sports on performance and injury prevalence. The present study has shown that comprehensive static stretching training can increase MVIC in longer muscle lengths, and hence might be a further training option for overhead sports athletes. Nevertheless, it would be important for future studies to address the effects of (different) interventions on the individual parts of the shoulder complex in order to develop a more comprehensive knowledge about the mechanisms behind functional and structural changes in relation to possible assistance with pain syndromes or as injury prevention.

There are some limitations coming along with this study. First, the ROM of the shoulder joint is also determined by the rotator cuff muscles [48]. These muscles are located in a deeper layer, and therefore, a measurement of their muscle stiffness was not feasible. Still, tissue stiffness of other muscles such as the rotator cuff muscles should be measured in future studies which would allow a better understanding of the mechanism of a long-term stretch training in the pectoralis muscle. Second, the control group has performed stretching and foam rolling exercises of the lower limbs and hence, a potential cross-over training effect in ROM as has been seen after a single bout of stretching [49] or foam rolling [50] cannot be ruled out completely. Third, information about other potential structural/architectural changes within the pectoralis major muscle such as fascicle length or muscle thickness would have been beneficial for our study. However, these measurements were not conducted, as an extrapolation of curved and fusiformis-like fascicles of the PMa pars clavicularis would have been too imprecise. Additionally, for the assessment of muscle thickness, it is recommended to use MRI measurements instead of ultrasound [51], which, however, was not performed in this study. For a better and comprehensive interpretation of long-term stretch training, it is therefore strongly recommended to include MRI measurements to assess muscle thickness.

## Conclusions

Similar to studies on long-term static stretching training of the lower limbs, we found that PMa muscle stretching increases shoulder extension ROM. In addition, this study showed improved maximum active torque, at long but not short muscle lengths. This indicates an increase in fascicle length, and hence a potential increase in sarcomeres in series. Furthermore, it can be assumed that either pain tolerance and/or changes in the stiffness of structures other than the muscles (e.g., ligaments, tendons, capsules) are responsible for the changes in ROM and active torque. Sports that require extended movement in the shoulder joint (i.e., overhead sports), in particular, and activities of daily living with extensive movements in the shoulder joint could benefit from the increase in flexibility and strength in the lengthened muscle following comprehensive static stretching training.

## Data Availability

All data will be made available on request to the corresponding author.

## Abbreviations

CG: Control group
CV: Coefficient of variance
ICC: Intraclass correlation coefficient
MVIC: Maximal voluntary isometric contraction
PMa: Pectoralis major muscle
PMa-SS: Pectoralis major static stretching intervention
ROI: Region of interest
ROM: Range of motion
sEMG: Surface electromyography
SWE: Shear wave elastography
